# Structural, Magnetic and Catalytic Properties of a New Vacancy Ordered Perovskite Type Barium Cobaltate BaCoO_2.67_

**DOI:** 10.1002/chem.202101167

**Published:** 2021-06-01

**Authors:** Aamir Iqbal Waidha, Humera Khatoon Siddiqui, Yuji Ikeda, Maren Lepple, Sami Vasala, Manuel Donzelli, A. D. Fortes, Peter Slater, Blazej Grabowski, Ulrike I. Kramm, Oliver Clemens

**Affiliations:** ^1^ Materials Synthesis Group Institute of Material Science University of Stuttgart Hesisenbergstraße 3 70569 Stuttgart Germany; ^2^ Catalysts and Electrocatalyst Department of Chemistry Eduard-Zintl Institute for Inorganic and Physical Chemistry Technische Universität Darmstadt 64287 Darmstadt Germany; ^3^ Department of Materials Design Institute for Materials Science University of Stuttgart Pfaffenwaldring 55 70569 Stuttgart Germany; ^4^ DECHEMA-Forschungsinstitut Theodor-Heuss-Allee 25 60486 Frankfurt am Main Germany; ^5^ Rutherford Appleton Laboratory Harwell Science and Innovation Campus ISIS Facility Didcot Oxfordshire OX11 0QX UK; ^6^ School of Chemistry University of Birmingham Edgbaston Birmingham B15 2TT UK

**Keywords:** ab initio calculations, bifunctional catalyst, magnetism, neutron diffraction, perovskite

## Abstract

A new vacancy ordered, anion deficient perovskite modification with composition of BaCoO_2.67_ (Ba_3_Co_3_O_8_□_1_) has been prepared via a two‐step heating process. Combined Rietveld analysis of neutron and X‐ray powder diffraction data shows a novel ordering of oxygen vacancies not known before for barium cobaltates. A combination of neutron powder diffraction, magnetic measurements, and density functional theory (DFT) studies confirms G‐type antiferromagnetic ordering. From impedance measurements, the electronic conductivity of the order of 10^−4^ S cm^−1^ is determined. Remarkably, the bifunctional catalytic activity for oxygen evolution reaction (OER) and oxygen reduction reaction (ORR) is found to be comparable to that of Ba_0.5_Sr_0.5_Co_0.8_Fe_0.2_O_3–*y*_, confirming that charge‐ordered anion deficient non‐cubic perovskites can be highly efficient catalysts.

## Graphical abstract

**A new charge ordered oxygen** deficient perovskite in barium cobaltate system is reported with bifunctional catalytic activity for oxygen reduction and oxygen evolution comparable to that to that benchmark perovskite catalyst BSCF.

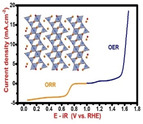

Perovskite‐type *ABX*_3‐*y*_ compounds have received a lot of attention due to their application in the fields of magnetism, fuel cells, solar cells and batteries.^[1]^ The ideal perovskite structure (*ABX*_3_) can be described as a cubic close packed (*ccp)* arrangement of *AX_3_* layers with *B* sites occupying 1/4 of octahedral voids resulting in a corner sharing octahedral network. The anion sublattice in such a structure is highly flexible and can accommodate a large amount of vacancies (*ABX*_3–*y,*_ *y*=1 being reported^[2]^), which can result in enhanced electronic conductivity due to mixed valency of the *B* site cation, which forms an important prerequisite towards developing perovskite catalysts for OER/ORR for fuel cell applications.^[3]^ In this respect, cobalt‐containing perovskites have been of particular interest due to their catalytic, magnetic and electronic properties.[^3^, ^4^]

For BaCoO_3‐*y*_ systems, a variety of compounds have been reported and summarized depending on the value of *y* by Raveau et al.^[1]^ and Mentré et al.^[5]^ For oxygen rich systems, i. e. for low values of *y*, hexagonal modifications (2H, 5H, 12H) are known and for higher values of *y*, the cubic modification or *ccp‐*related vacancy‐ordered variants are known (BaCoO_2.22_ and BaCoO_2_),[^2^, ^5^, ^6^] and *ccp*‐related arrangements can also be stabilized by the uptake of water (BaCoO_1.80_(OH)_0.86_).^[7]^ Such or similar barium cobaltates have attracted a lot of attention for their activity for the oxygen evolution reaction (OER).^[8]^

Herein, we report a new vacancy ordered *ccp*‐related modification of barium cobaltate with a composition of BaCoO_2.67_ (Ba_3_Co_3_O_8_□_1_, □=anion vacancy) prepared by a two‐step heating process together with its electrocatalytic and magnetic properties. The heating steps, times and temperatures were found to be of significant importance for the successful synthesis of the title compound. After the first heating step at 1273 K for 60 h under a flow of argon gas, X‐ray powder diffraction data indicate the formation of a barium cobaltate phase with a diffraction pattern similar to the vacancy‐ordered monoclinic modification of BaFeO_2.5_ ^[9]^ together with an additional tetragonal perovskite type phase (see Figure S1 in Supporting Information). Unfortunately, this phase could not be obtained in a pure form. From the energy dispersive X‐ray spectroscopical analysis (EDX), a 1 : 1 ratio of Ba : Co was confirmed (Figure S2 in Supporting Information), and iodometric titrations on this phase mixture indicated an average oxidation state of +2.93(1) for cobalt, implying an overall average composition of BaCoO_2.46_. In order to determine the topochemical oxidation behavior of this compound and suitable reaction conditions for oxygen uptake, simultaneous thermal analysis (STA, see Figure S3 in Supporting Information) was carried out in oxygen atmosphere, which showed an onset of oxygen uptake at 455 K. This mass increase corresponds to an uptake of ∼0.21 moles of oxygen, which means an average oxidation state of 3.34 for cobalt and a composition corresponding to ∼BaCoO_2.67_. Iodometric titrations confirmed this composition. At 535 K a second mass increase and oxygen uptake is observed, which would lead to a final composition of BaCoO_2.80_. The underlying phase changes were investigated by temperature dependent XRD measurements starting from the phase mixture with composition of BaCoO_2.46_ obtained after the first heating step in oxygen atmosphere. Changes in diffraction pattern are observed starting at 448 K (Figure S4a in Supporting Information). At 473 K, the X‐ray diffractogram shows the appearance of a single phase of the title compound (Figure S4b in Supporting Information), whereas the patterns recorded at 573 K and above show the presence of a hexagonal‐type modification (Figure S4c in Supporting Information), which agrees well with the further oxygen uptake observed in STA. The appearance of this hexagonal phase is in close agreement to what was reported previously by Raveau et al.^[1]^

To determine the structure and phase composition of the new modification with composition BaCoO_2.67_, a larger batch of this material was prepared from the as‐determined optimized reaction conditions of the second heating step and X‐ray diffraction as well as neutron diffraction data were recorded and analyzed, see Figure 1. The diffraction pattern showed principle similarity to the cubic perovskite modification with apparent splitting of the main reflections and appearance of superstructure reflections. All the reflections could be indexed with a monoclinic unit cell with *a*=10.1718(3) Å, *b*=5.6035(2) Å, *c*=6.9248(2) Å and β=91.465(4)°, where the lengths of the axes indicate a $\sqrt{6}$ ×$\sqrt{2}$ ×$\sqrt{3}$ supercell of the cubic aristotype structure (we note that ∼3.23(1) wt % of CoO were found as impurity for the larger batch, which indicates close to full homogeneity of the powder). Pawley fits indicated the centrosymmetric monoclinic space group *P*2_1_/*m* as the most likely symmetry in agreement with the missing (0 1 0) reflection, which is forbidden and could be observed neither in the X‐ray nor the neutron diffraction data, further indicating the presence of the 2_1_ screw axis (see Figure S5 in Supporting Information for the structural relationship). This symmetry had been previously found for the compound BaFeO_2.33_F_0.33_[^10^, ^11^] with similar overall anion composition by our group for which the symmetry lowering was found to originate from ordering of the anion vacancies. Another member, BaFeO_2.67_, of the Ba(Fe,Co)(O,F)_2.67_ family was recently prepared, and it appears to be isotypic to both BaFeO_2.33_F_0.33_ and BaCoO_2.67_, which we will report in a separate article. The structural similarity between these compounds is plausible since they contain similar overall anion contents showing that in the setting of a barium‐rich anion‐deficient perovskite lattice, only certain anion compositions can give highly favorable structural stabilization and in agreement with our previous finding that hydrated barium ferrates and cobaltates possess close structural similarity.[^2^, ^3^, ^7^, ^12^] Therefore, the structural model of BaFeO_2.33_F_0.33_ suggested by Clemens et al.^[11]^ was used as the starting model (see Table S1 in Supporting Information for the starting model) for the structural analysis of the X‐ray and neutron diffraction data. Relaxation of the model resulted in an excellent fitting of the diffraction data with chemically plausible structural model (see bond distances in Table S5 provided in Supporting Information). Similar to BaFeO_2.33_F_0.33_, the 2*a* anion site was found to be vacant which, in combination with smaller structural relaxations, gives rise to cobalt being present in three different coordination environments, i. e., tetrahedral, square pyramidal and octahedral (see Table S2 in Supporting Information for the refined structural model and Figure 2 structural schematics). Compared to BaFeO_2.33_F_0.33,_ wherein the Fe cations are only present in the trivalent oxidation state, BaCoO_2.67_ contains mixed valent B site as determined from iodometic titrations corresponding to +3 and +4 oxidation states. From the structural refinements, the average Co−O distances of 2.02 Å, 1.79 Å and 2.01 Å for 6‐, 5‐ and 4‐fold coordination were determined for Co1, Co2 and Co3 respectively, which indicates an oxidation state of +3 for Co1 and Co3, as well as +4 for the Co2 site from a consideration of average bond distance,^[13]^ and a calculation of bond valence sums (see Table S6 in Supporting Information). We also would like to acknowledge that we attempted to refine the structure in non‐centrosymmetric monoclinic subgroups or within the triclinic subgroup *P‐*1. Since these models did not result in any significant improvement of the fit, they were discarded.

**Figure 1:**
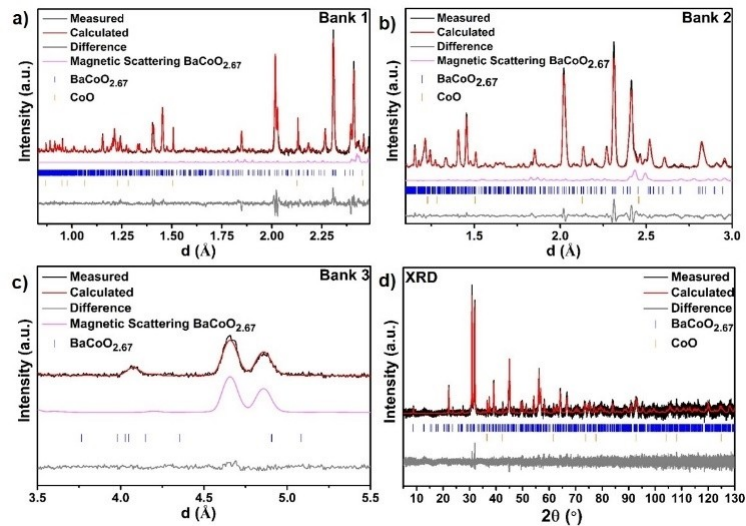
Coupled Rietveld analysis of the room temperature neutron (HRPD, bank 1–3, a–c) and X‐ray diffraction (d) patters recorded for BaCoO_2.67_.

**Figure 2:**
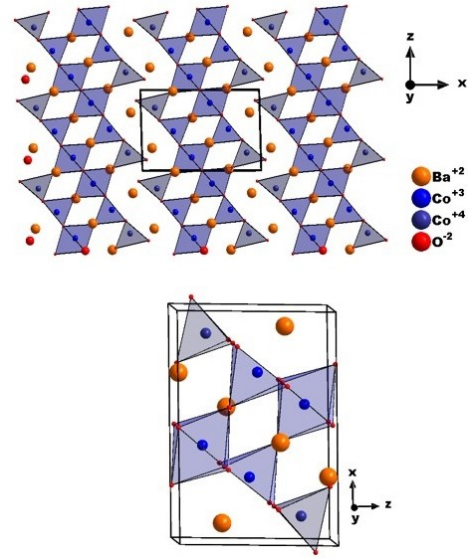
Refined crystal structure of BaCoO_2.67_ obtained from the coupled Rietveld fit of neutron and X‐ray diffraction data showing the vacancy ordering and three different Co coordination environments.

Field dependent measurements of magnetization (M−H) were recorded at 10, 80, 250 and 320 K (Figure 3a), which mainly indicated an antiferromagnetic behavior with only a small residual ferromagnetic moment at 10 K (∼0.00003 μ_B_). This is in agreement with field and zero field cooled measurements (Figure 3b) which showed a small but significant difference. This ferromagnetic contribution could be due to the presence of impurity or magnetic canting, which would be undeterminable via powder diffraction techniques. The antiferromagnetic ordering below 350 K is further indicated by the presence of magnetic reflections in the neutron powder diffraction patterns measured at ambient temperatures. These magnetic reflections indicate a magnetic *k*‐vector of [0 0 1/2 ]. Thus we attempted to refine the magnetic intensity with different structural models, and found that it can be best described with a G‐type antiferromagnetic structure with an average magnetic moment of 2.36(1) μ_B_ aligned along the *c*‐axis. The magnetic moment obtained from the refinements suggests an intermediate spin state for Co with ∼1–2 unpaired electrons on average. Density functional theory (DFT) based calculations also found consistently that the G‐type antiferromagnetically ordered state (G‐AFM) is energetically more favorable than the ferromagnetic (FM) state, which nicely supports the experiments above (Figure S6 in Supporting Information).

**Figure 3:**
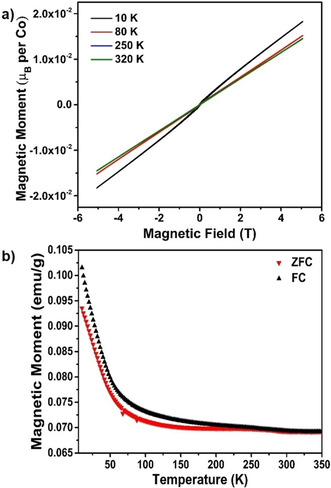
a) Field dependent measurements of magnetization of BaCoO_2.67_. b) Temperature dependent measurements of magnetization for zero‐field cooled and field‐cooled sample of BaCoO_2.67_.

Electrochemical impedance spectroscopy measurements (Figure S7 in Supporting Information) were carried out to determine the conductivity of the sample. From the NYQUIST plot a single depressed semicircle was observed which was fitted using a single R‐CPE (constant phase element). The room temperature conductivity of the sample was determined from the intercept on the X‐axis and calculated to be 2.89×10^−4^ S cm^−1^ which is of the same order of magnitude as that of BaCoO_1.80_(OH)_0.86_ ^[7]^ and three orders of magnitude higher than that of BaCoO_2+δ_.^[2]^ This is in agreement with what is expected from the presence of Co in a mixed oxidation state (+3/+4). An activation energy of 0.21(1) eV was calculated from the slope of the Arrhenius plot (Figure 4a) indicating the dominance of electronic contribution to overall conductivity.^[2]^

**Figure 4:**
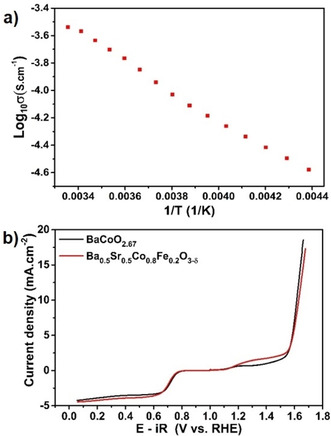
a) Arrhenius plot for BaCoO_2.67_ measured in the temperature range of 228 to 298 K. b) Comparison of ORR/OER activity between BaCoO_2.67_ and BSCF in 0.1 M KOH. For ORR and OER the cathodic and anodic sweep are displayed, respectively.

The catalytic activity for OER and ORR was measured for the title compound BaCoO_2.67_ and compared to that of Ba_0.5_Sr_0.5_Co_0.8_Fe_0.2_O_3–*y*_ (BSCF) which is the benchmark perovskite catalyst[^4^, ^14^] (see section 4 and Figures S8–S11 in Supporting Information for more details on preparation of reference electrodes and comparative morphological as well as electrochemical characterization). The catalytic activity in the absence of carbon is low for both compounds (see Figures S10 and S11). Referencing the activity data to the surface area of the perovskites (Figure S10c and d) indicate similar onset of both materials for the ORR while the overall current density (per BET surface area) is larger for Ba_0.5_Sr_0.5_Co_0.8_Fe_0.2_O_3‐*y*_. The performance towards OER is much better for Ba_0.5_Sr_0.5_Co_0.8_Fe_0.2_O_3‐*y*_ which might be related to the integration of iron in the system, as it is known for several OER catalysts.^[15]^

Once carbon is added, the catalytic activity of the title compound is very similar to that of the BSCF (for reasons of comparability, the OER and ORR data are plotted together and are shown in Figure 4b), though the BaCoO_2.67_ particles showed a much coarser crystallinity (see Figure S9 in Supporting Information). From the ORR region, we see that the potential of 0.7 V vs. RHE (reversible hydrogen electrode) a kinetic current density of 2.3 mA cm^−2^ is achieved and an onset potential of 0.81 V for BaCoO_2.67_. On the other hand, BSCF shows a kinetic current density of 2.1 mA cm^−2^ at 0.7 V with the onset potential of 0.785 V. The positive shift of the onset potential and larger current density therefore indicate a slightly better ORR activity for BaCoO_2.67_ as compared to BSCF.

In addition to ORR we also investigated the OER characteristics of both BaCoO_2.67_ and BSCF. With the addition of carbon both compounds show almost similar onset potentials and achieve the benchmarking value of 10 mA cm^−2^ at similar potential. The Tafel slopes of both samples are higher in comparison to literature values^[16]^ (see Figure S12 and Table S7 in Supporting Information). The origin of this, might be found in smaller conductivity or a larger grain size of our samples in comparison to those investigated in the literature. The bifunctional performance (Δ*U*=*U*_OER_–*U*_ORR_) was calculated to be 0.95 V for both BaCoO_2.67_ and BSCF which is much lower compared to the BaFe_1–*x*_Co_*x*_O_3–*y*–δ_(OH)_*y*_ series^[3]^ and comparable to that of noble metal catalyst.^[17]^

In relation to the suggested dependency of the catalytic activity on electron configuration as provided by Suntivich et al.,[^14^, ^18^] we aim to provide a qualitative reasoning why similarly high activity can be obtained for BaCoO_2.67_ in comparison to BSCF, though no morphological optimization of the compound was performed. According to Suntivich et al.[^14^, ^18^] both ORR and OER activity are highly dependent on the e_g_ orbital filling of the transition metal ion with an average occupation of one electron being desirable. For mixed‐valent BaCoO_2.67_, all Co ions are located in polyhedra with a local site symmetry of *m*. Such an average filling of e_g_ states is plausible if Co is present in the intermediate spin state. This would result in the electronic configuration of t_2g_^5^e_g_^1^ for Co^3+^ (and high spin e_g_^3^t_2g_^2^ for Co^4+^), which could be brought in principle agreement with the local symmetry and thus non‐degeneracy within e_g_/t_2g_ levels,^[1]^ and which might favor such similar catalytic activity. For BSCF, Co has been reported to be present in the intermediate state which would result in the likely electronic configuration of t_2g_^5^e_g_∼^1.25^ (average Co oxidation state of +2.75 determined from iodometric titrations).^[14]^ In addition to the impact of the ordered coordinationenvironments and their impact on the electronic configurations, this results in a high density of states (DOS) near the Fermi level for both BSCF and BaCoO_2.67_ (Figure S11). Although determining the exact spin state of Co in BaCoO_2.67_ on the different crystallographic sites with octahedral, square pyramidal, or tetragonal coordination is hindered experimentally, the catalytic activity observed here in combination with the magnetic moment observed at room temperature by neutron diffraction is plausible in relation to electron configurations found in similar compounds, with an overall occupation of the higher *d*‐orbital energy levels by 1.33 electrons for BaCoO_2.67_. Thus, ordering of vacancies can be considered to be the origin for the resulting ordering of charges and electron configurations, which in turn contribute to achieve a high catalytic activity in BaCoO_2.67._

## Conflict of interest

The authors declare no conflict of interest.

## Supporting information

As a service to our authors and readers, this journal provides supporting information supplied by the authors. Such materials are peer reviewed and may be re‐organized for online delivery, but are not copy‐edited or typeset. Technical support issues arising from supporting information (other than missing files) should be addressed to the authors.

Supplementary

## References

[1] B. Raveau, M. Seikh, Cobalt Oxides: From Crystal Chemistry to Physics, Wiley VCH, Weinheim, 2012, p. 3–70.

[2] A. I. Waidha, H. Zhang, M. Lepple, S. Dasgupta, L. Alff, P. Slater, A. D. Fortes, O. Clemens, Chem. Commun. 2019, 55, 2920–2923. 10.1039/c8cc09532a 30762042

[3] A. I. Waidha, L. Ni, J. Ali, M. Lepple, M. Donzelli, S. Dasgupta, S. Wollstadt, L. Alff, U. I. Kramm, O. Clemens, J. Mater. Chem. A 2020, 8, 616–625.

[4] G. Chen, W. Zhou, D. Guan, J. Sunarso, Y. Zhu, X. Hu, W. Zhang, Z. Shao, Sci. Adv. 2017, 3, e1603206. 28691090 10.1126/sciadv.1603206 PMC5479656

[5] O. Mentre, M. Iorgulescu, M. Huve, H. Kabbour, N. Renaut, S. Daviero-Minaud, S. Colis, P. Roussel, Dalton Trans. 2015, 44, 10728–10737. 25683340 10.1039/c4dt03874f

[6] U. Spitsbergen, Acta Crystallogr. 1960, 13, 197–198.

[7] A. I. Waidha, M. Lepple, K. Wissel, A. Benes, S. Wollstadt, P. R. Slater, A. D. Fortes, O. Clemens, Dalton Trans. 2018, 47, 11136–11145. 30043789 10.1039/c8dt01326h

[8a] X. Xu, C. Su, W. Zhou, Y. Zhu, Y. Chen, Z. Shao, Adv. Sci. 2016, 3, 1500187; 10.1002/advs.201500187 PMC5054898 27774387

[8b] X. Xu, W. Wang, W. Zhou, Z. Shao, Small Methods 2018, 2,1800071 –.

[9] O. Clemens, M. Groting, R. Witte, J. M. Perez-Mato, C. Loho, F. J. Berry, R. Kruk, K. S. Knight, A. J. Wright, H. Hahn, P. R. Slater, Inorg. Chem. 2014, 53, 5911–5921. 24901981 10.1021/ic402988y

[10] O. Clemens, J. Solid State Chem. 2015, 225, 261–270.

[11] O. Clemens, C. Reitz, R. Witte, R. Kruk, R. I. Smith, J. Solid State Chem. 2016, 243, 31–37.

[12] T. Yamamoto, Y. Kobayashi, N. Hayashi, C. Tassel, T. Saito, S. Yamanaka, M. Takano, K. Ohoyama, Y. Shimakawa, K. Yoshimura, H. Kageyama, J. Am. Chem. Soc. 2012, 134, 11444–11454. 22708676 10.1021/ja3007403

[13] R. D. Shannon, Acta Crystallogr. Sect. A 1976, A32, 751–767.

[14] J. Suntivich, K. J. May, H. A. Gasteiger, J. B. Goodenough, Y. Shao-Horn, Science 2011, 334, 1383–1385. 22033519 10.1126/science.1212858

[15a] N. Weidler, S. Paulus, J. Schuch, J. Klett, S. Hoch, P. Stenner, A. Maljusch, J. Brotz, C. Wittich, B. Kaiser, W. Jaegermann, Phys. Chem. Chem. Phys. 2016, 18, 10708–10718; 26694730 10.1039/c5cp05691h

[15b] D. Friebel, M. W. Louie, M. Bajdich, K. E. Sanwald, Y. Cai, A. M. Wise, M. J. Cheng, D. Sokaras, T. C. Weng, R. Alonso-Mori, R. C. Davis, J. R. Bargar, J. K. Norskov, A. Nilsson, A. T. Bell, J. Am. Chem. Soc. 2015, 137, 1305–1313; 25562406 10.1021/ja511559d

[15c] J. Schuch, S. Klemenz, P. Schuldt, A. M. Zieschang, S. Dolique, P. Connor, B. Kaiser, U. I. Kramm, B. Albert, W. Jaegermann, ChemCatChem 2021, 13, 1772–1780.

[16a] R. Mohamed, X. Cheng, E. Fabbri, P. Levecque, R. Kötz, O. Conrad, T. J. Schmidt, J. Electrochem. Soc. 2015, 162, F579–F586;

[16b] Y. Zhu, W. Zhou, J. Yu, Y. Chen, M. Liu, Z. Shao, Chem. Mater. 2016, 28, 1691–1697.

[17] Y. Gorlin, T. F. Jaramillo, J. Am. Chem. Soc. 2010, 132, 13612–13614. 20839797 10.1021/ja104587v

[18] J. Suntivich, H. A. Gasteiger, N. Yabuuchi, H. Nakanishi, J. B. Goodenough, Y. Shao-Horn, Nat. Chem. 2011, 3, 546–550. 21697876 10.1038/nchem.1069

