# Supplementary material for: Structural, Magnetic and Catalytic Properties of a New Vacancy Ordered Perovskite Type Barium Cobaltate BaCoO2.67
Source: Chemistry. 2021 Jun 1;27(38):9763–7. doi: 10.1002/chem.202101167 (PMC8361746; doi:10.1002/chem.202101167)
Supplement: Supplementary file 1 — Supplementary [file CHEM-27-9763-s001.pdf]

Chemistry–A European Journal

Supporting Information

Structural, Magnetic and Catalytic Properties of a New Vacancy Ordered Perovskite Type Barium Cobaltate $BaCoO_{2.67}$

Aamir Iqbal Waidha, Humera Khatoon Siddiqui, Yuji Ikeda, Maren Lepple, Sami Vasala, Manuel Donzelli, A. D. Fortes, Peter Slater, Blazej Grabowski, Ulrike I. Kramm, and Oliver Clemens*

1 Experimental Procedures

1.1 Material synthesis

Conventional high temperature solid state synthesis was used for the synthesis of the intermediate phase. Precursor powders of $BaCO_3$ (99.99%, Alfa Aesar) and Co_3O_4 (99.99%, Alfa Aesar) were hand milled (with acetone) followed by heating in a tube furnace up to 1273 K for 60 hours with a heating and cooling rate of 2 K per minute under argon atmosphere. The as obtained powder was then hand milled and heated to 473 K under oxygen atmosphere for 2 hours. After the second cycle the desired phase was obtained.

Powders with chemical composition $Ba_{0.5}Sr_{0.5}Co_{0.8}Fe_{0.2}O_{3-\delta}$ (BSCF) were synthesized using nebulized spray pyrolysis (NSP). To prepare the starting precursor solution, $Ba(NO_3)_2$ (Sigma Aldrich, 99.99%), $Sr(NO_3)_2$ (Sigma Aldrich, 99.99%), $Fe(NO_3)_3(H_2O)_9$ (Sigma Aldrich, 99.99%) and $Co(NO_3)_2(H_2O)_6$ (Sigma Aldrich, 99.99%) were dissolved in de-ionized water to obtain an overall cation concentration of 0.06 mol l^{-1}, and the mixture was stirred for 30 minutes in order to obtain a homogenous transparent solution. For the synthesis, a setup as previously described was used [1]. The as-prepared solution was injected into the nebulizer unit using a syringe pump with a flow rate of 1.5 ml min^{-1}, while operating the ultrasonicator to generate the mist at a generator voltage and current of 47.0 V and 0.51 A respectively. The precursor mist formed inside the nebulizer unit was transported by a carrier gas stream of argon at the rate of 2 SLM (standard liter per minute) to the reaction tube, which was maintained at 1323 K, and the compounds were collected on a glass filter collector which was held at 393 K. The whole process was carried out at a constant pressure of 900 mbar.

1.2 Diffraction experiments

X-ray diffraction (XRD) patterns of the compounds were recorded on a Bruker D8 diffractometer using Bragg-Brentano geometry with a fine focusing X-ray tube with Cu $K_{\alpha 1,}$ radiation. A VANTEC detector (3° opening) and a variable divergence slit (4 mm) were used. The total scan time was set to 10 hours for the angular range between 5° and 130° 2θ at a step size of 0.0075. High temperature XRD was measured using an Anton Paar HTK1200 sample stage in the temperature range between 303 K and 973 K (heating rate of 5 K s^{-1}) in the angular range between 20° and 60° 2θ with the step size of 0.0075° (total counting time of 10 min per scan) under a flow of oxygen. These conditions were chosen as a compromise to obtain sufficient data quality for the determination of lattice parameters and phase composition, while lowering differences to the heating procedure used for simultaneous thermal analysis.

Time-of-flight (TOF) neutron powder diffraction (NPD) data were recorded on the high resolution diffractometer (HRPD) at the ISIS pulsed spallation source (Rutherford Appleton Laboratory, Didcot, U.K.). 2 g of powdered $BaCoO_{2.67}$ was loaded into 6-mm-diameter thin-walled cylindrical vanadium sample can under an inert atmosphere and sealed with indium wire. Data were collected at ambient temperature for 2 h 40 m (120 µAh of integrated proton beam current) using the instrument's 30–130 ms time-of-flight window. Raw data were normalized to the incident spectrum and corrected for instrument efficiency using a V:Nb null-scattering standard with the Mantid suite of diffraction algorithms (http://www.mantidproject.org/).

The analysis of the nuclear structure of $BaCoO_{2.67}$ using both the NPD and XRD data was performed using the Rietveld method with the program TOPAS 5 (Bruker AXS, Karlsruhe, Germany) [2]. For the room temperature XRD data, the whole 2θ-range range was used, while for NPD, data collected from the highest resolution backscattering detector bank (bank 1, 158° < 2□ < 176°) as well as the high intensity bank (bank 2, 80° < 2□ < 100°) and the forward-scattering bank 3 (28° < 2□ < 32°) were used. For better depiction, the fit and measurement curves of the XRD pattern shown in Figure 2d in the manuscript were corrected by the refined background curve.

The instrumental intensity distribution for the X-ray data was determined empirically from a sort of fundamental parameters set [3], using a reference scan of LaB_6 (NIST 660a), and the microstructural parameters were refined to adjust the peak shapes for the XRD data. Calibration of the instrumental contribution to the neutron powder

diffraction pattern's peak positions and shapes was done using silicon and ceria standards. The lattice parameters were constrained to be the same for neutron and XRD data, and the same positional parameters were used and refined for both data sets. Independent thermal displacement parameters were refined for each type of atom for neutron data.

1.3 Simultaneous thermal analysis

Simultaneous thermal analysis combining thermogravimetric analysis (TGA) and differential scanning calorimetry (DSC) were conducted for $BaCoO_{2.67}$ on a Netzsch STA 449 F3 Jupiter thermal analyzer. The measurements were performed in the temperature range between 300 K to 973 K using a heating rate of 10 K min^{-1}. Thermal analysis was performed in corundum crucibles under an oxygen flow of 50 ml min^{-1}.

1.4 Impedance spectroscopy analysis

Alternating current electrochemical impedance spectroscopy measurements were carried out to record the conductivity of the $BaCoO_{2.67}$ and the BSCF pellet. The pellets were made by isotatic pressing of the powders of $BaCoO_{2.67}$ at 700 kN. The pellet was placed inside the JANIS STVP-200-XG cryostat, which was operated under static helium atmosphere of 1 bar pressure. The pellet was investigated in the temperature range of 298 K to 228 K. Impedance measurements were recorded using a Solartron 1260 frequency response analyzer while applying an AC signal of 100 mV amplitude with frequency ranging from 1 MHz to 100 mHz. Fitting of the data was performed using the Z-view program [4].

1.5 SQUID magnetic measurements

Magnetic characterization was performed with a Quantum Design MPMS. Powder samples were encapsulated in gelatin capsules and mounted in a straw. Zero-field cooled (ZFC) and field-cooled (FC) curves were measured from 5 to 350 K in the applied field $\mu_0H = 1$ T. All magnetization measurements were corrected by the diamagnetic contributions of the phases and of the gelatin capsule and straw used for sample mounting. Field-dependent measurements were performed at 5 K. The magnetic field was scanned from 5 to −5 T.

1.6 Scanning electron microscopy and Energy Dispersive X-ray spectroscopy

The scanning electron microscopy (SEM) images were taken using the secondary electron detector of a Philips XL30 FEG scanning electron microscope operating at 10 keV. The energy dispersive spectroscopy (EDAX) analysis was carried out using Phillips XL30 FEG set up operating at 15 keV. An EDAX Genesis system was used and an energy resolution of about 140 eV was applied. The mapped area was of the order of 100 μm^2 and the Ba to Co to Fe ratio was determined from the Ba L, Fe K and Co K lines. The samples were sputtered with approximately 10 nm of Au prior to the measurements.

1.7 Iodometric titration

The iodometric titrations were carried out to determine the oxidation state of the Co ions in the intermediate compound $BaCoO_{2.46}$, $BaCoO_{2.67}$. and BSCF. 0.05 g of the compound was dissolved in 1M HCl solution and 1 g of KI was added to the solution. Due to the presence of Co^{2+} ions no formation of I_2 could be observed. A few drops of starch solution were added as an indicator. The solution was titrated with sodium thiosulphate ($Na_2S_2O_3$).

1.8 X-ray photoelectron spectroscopy

The valence states were examined by ex-situ X-ray photoelectron spectroscopy (XPS) analysis using a Physical Electronic VersaProbe XPS unit (PHI 5000 spectrometer) with Al K_α radiation (1486.6 eV). All detailed spectra were

recorded with 50.6 W, a step size of 0.1 eV and a pass energy of 23.5 eV. The binding energies were calibrated with respect to the carbon 1s (C1s) emission line at 284.8 eV. Neutralizer was used for all the samples to account for the charging effects. For the determination of Cobalt oxidation states, the Co3p emission was selected since the Co2p and the Ba3d emission strongly overlap and a deconvolution of the signals is not possible. In order to ensure a better comparability, the valence band spectra were normalized to a uniform maximum intensity.

1.9 N_2 sorption Measurements

N_2 sorption measurements were performed with an Autosorb 3 instrument (Quantachrome). In a first step samples were degassed at 200 °C overnight to remove possibly adsorbed species. Afterwards the samples were transferred to the measuring station. The nitrogen adsorption and desorption isotherms were recorded and the BET specific surface area determined.

1.10 Electrochemical Measurements

The electrocatalytic activity of these perovskite-type $BaCoO_{2.67}$ and BSCF catalysts towards OER and ORR was investigated using a conventional three electrode setup with a Hg/HgO reference electrode and glassy carbon rod counter electrode. The samples were deposited on the glassy carbon disc of a rotating disc electrode (RDE, PINE) that was connected to a Parstat 3F potentiostat (Versastat). The glassy carbon (GC) electrode was first polished with 1 Fµm diamond on a micro cloth, followed by a 50 nm Al_2O_3 on a master tex polishing cloth, ending by sonicating and rinsing in ethanol and distilled water, respectively. For the preparation of the ink, 5 mg of the sample was mixed with 1.66 mg of carbon black (P-XP, PentaCarbon GmbH, Germany) as a conductive additive and to enhance the utilization of the perovskite. 25 µl of a Nafion solution (5 wt %, QUINTECH) and 83.3 µl of isopropanol and 142 µl of distilled water were added before the suspension was treated firstly by vortex for several seconds and then in an ultrasonic bath for 60 min, ending with the dispersion by vortex to get a homogeneous ink. Afterwards, 6µl of the freshly prepared ink was deposited on the surface of the glassy carbon tip of the RDE electrode (geometrical area of 0.2375 cm^2) and the catalyst loading was 0.5 mg cm^{-2} (carbon not considered). Reference measurements of the pure materials (without carbon black addition) were made for reasons of comparison. Ink preparation and catalyst loading were kept the same.

The electrolyte was 0.1M KOH solution (Potassium hydroxide, >99%, Sigma-Aldrich) saturated with N_2 and O_2 for the OER and ORR, respectively. The electrochemical activity towards OER and ORR was evaluated by cyclic voltammetry (CV) at a rotation speed of 1600 rpm from 1.0 to 1.9 V (OER) and from 0.0 to 1.0 V (ORR) versus reversible hydrogen electrode (RHE) both with a sweep rate of 10 mV s^{-1}. Before the electrochemical tests, an activation step was applied to electrochemically activate the materials and remove the bubbles inside of the catalyst layer by running CV in a range of 1.0 to 0 V_{RHE} for 20 cycles with 100 mV s^{-1} until the observed current voltage characteristic did not change anymore. It should be pointed out that all the potential data are reported applying an *iR* correction ($E_{iR-\text{corrected}} = E - iR$) by compensating for the solution resistance.
The ORR data were capacity corrected by subtracting the CV data obtained in the same potential window and with the same sweep rate but in N_2 saturated electrolyte. For ORR the cathodic sweep and for OER the anodic sweep are displayed.
For reasons of comparison, onset potentials were determined. As Onset potential for ORR we define the potential where the current density becomes lower than -0.1 mA cm^{-2}. Similarly, the onset potential for OER is defined as that potential where the current density becomes larger than 0.1 mA cm^{-2}.

2 Computational Details

The oxygen-vacancy-ordered $P2_1/m$ $Ba_3Co_3O_8$ = $BaCoO_{2.67}$ structure has a 28-atom primitive cell. To be commensurate with the G-type antiferromagnetic (AFM) ordering (in the Wollan–Koehler notation [5]) a 1 × 1 × 2 supercell including 56 atoms was employed as the simulation cell.

We employed the projector augmented wave method [6] and the generalized gradient approximation (GGA) of the Perdew–Burke–Ernzerhof (PBE) form [7] implemented in the VASP code [8] in combination with the provided potentials [9]. The 5s5p6s, 3d4s, and 2s2p orbitals of Ba, Co, and O, respectively, were treated as valence states. The plane-wave cutoff was set to 550 eV. The reciprocal space of the 56-atom supercell was sampled by a Γ-centered 6 × 8 × 4 mesh, and the Methfessel–Paxton scheme [10] with a smearing width of 0.1 eV was employed. Total energies were minimized until they were converged to within 10^{-5} eV per unit cell. Cell volume, cell shape, and internal atomic positions were optimized so that the forces on atoms and the stress components on the supercells are less than 2.5×10^{-2} eV/Å and 10^{-3} eV/Å^3, respectively. In addition to the G-AFM state, the nonmagnetic (NM) and the ferromagnetic (FM) states were also computed under the collinear spin-polarized condition. Strong on-site electron correlation was considered for the Co d orbitals employing the rotationally invariant DFT+U formalism of Dudarev *et al.*[11] with $U_{eff} \equiv U - J$ varied in a range of 0–6 eV.

As found in Figure S6, for the considered U_{eff} range, the G-AFM state is energetically more stable than the FM state, consistently with the experiments in the present study. Following a previous study [12], we focus on $U_{eff} = U - J = 4.5$ eV. The internal atomic positions and lattice parameters are summarized in Table S3 and Table S4, respectively, which show reasonable agreement with experimental values (Table S2).

3 Results and Discussion

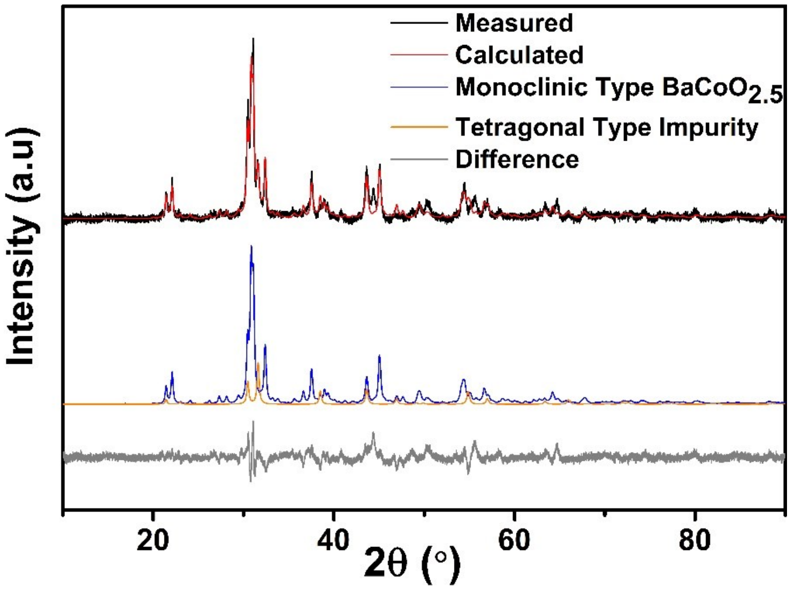

Figure S1:Rietveld refinement of intermediate solid state synthesized powder containing a $BaFeO_{2.5}$ type phase and an unknown perovskite related second phase (roughly described here with a tetragonal distorted perovskite phase).

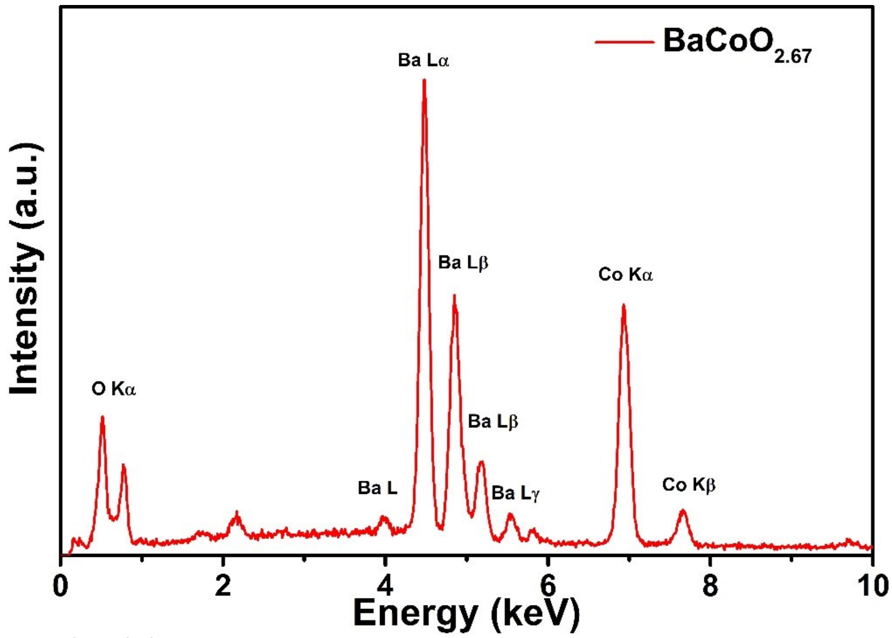

Figure S2: EDAX spectra recorded for $BaCoO_{2.67}$ powders using 10 keV electron beam.

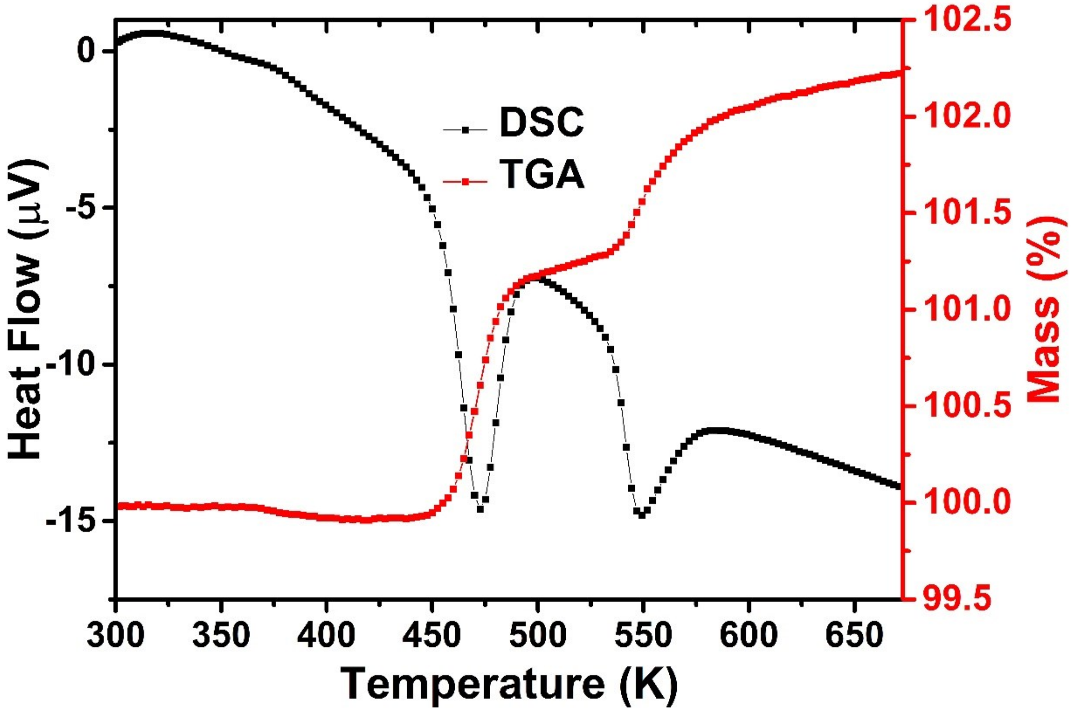

Figure S3: TGA and DSC curve measured under the flow of O_2 on the intermediate compound $BaCoO_{2.46}$.

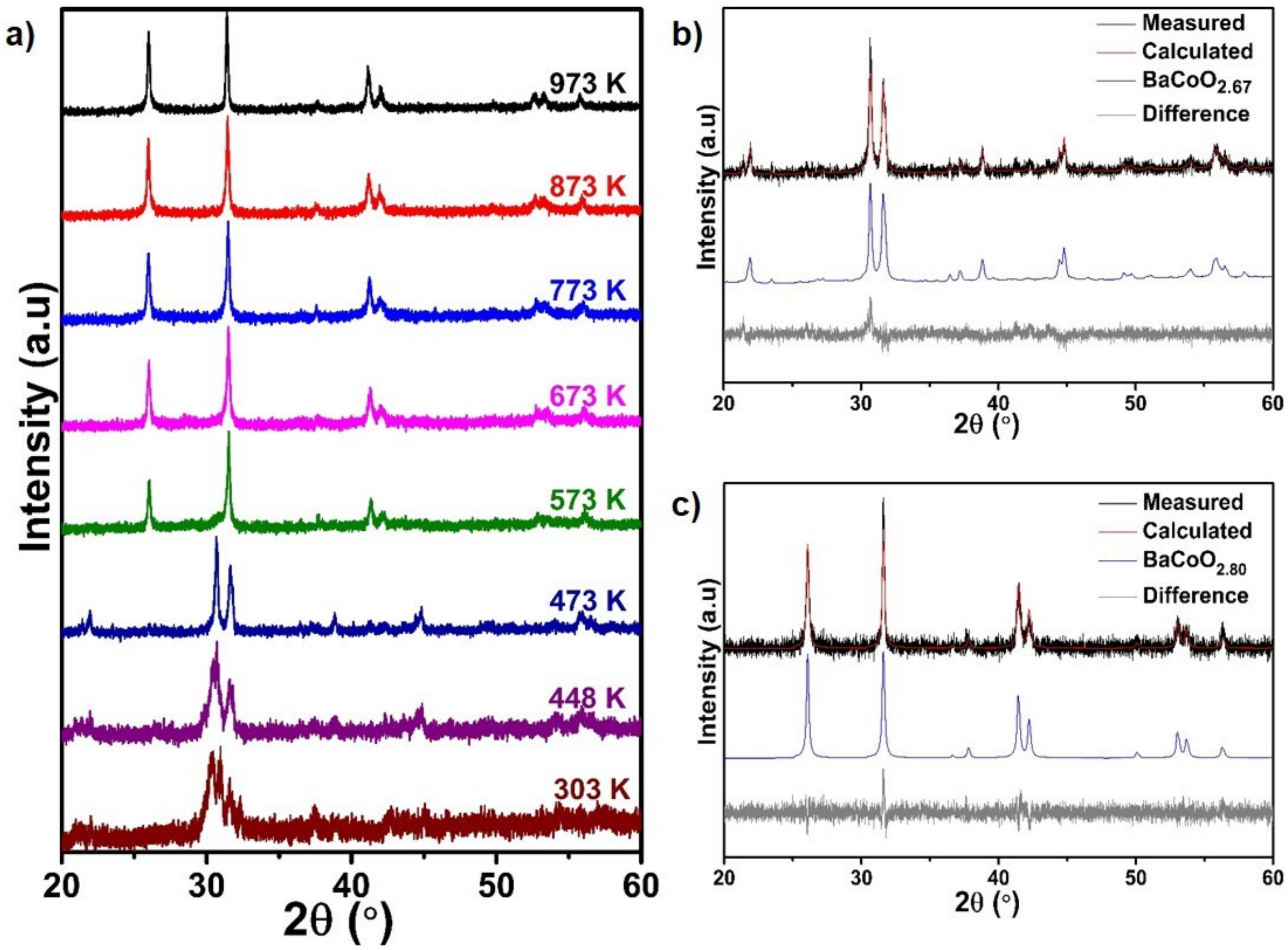

Figure S4*:* a) High temperature XRD diffractogram for the powder starting from $BaCoO_{2.46}$ powder measured under oxygen atmosphere. b)&c) Rietveld refinement of the diffractogram measured at 473 K and 973 K respectively.

Cubic
Pm-3*m* (221)
a_1 = 4.06 Å

Ba: 1*a*<br>*m*-3*m*	Co: 1*b*<br>*m*-3*m*	O: 3*c*<br>4/*mm.m*
0<br>0<br>0	½<br>½<br>½	0<br>½<br>½

↓ *t*4: $a_2 = a_1 - b_1$, $b_2 = b_1 - c_1$, $c_2 = a_1 + b_1 + c_1$

Trigonal
R-3*m* (166)
a_2 = 5.74 Å
b_2 = 5.74 Å
c_2 = 7.03 Å

Ba: 3*a*<br>-3*m*	Co: 3b<br>-3*m*	O: 9*e*<br>.2/*m*
0<br>0<br>0	½<br>½<br>½	½<br>0<br>0

↓ *k*2: $a_3 = a_2$, $b_3 = b_2$, $c_3 = c_2$

Trigonal
P-3*m* (164)
a_3 = 5.74 Å
b_3 = 5.74 Å
c_3 = 7.03 Å

Ba: 1*a*<br>-3*m*	Ba: 2*d*<br>3*m.*	Co: 1*b*<br>-3*m.*	Co: 2*d*<br>-3*m.*	O: 3*e*<br>.2/*m.*	O: 6*i*<br>.*m.*
0<br>0<br>0	½<br>⅔<br>*z*<br>*z* ~0.667	0<br>0<br>½	0<br>⅔<br>*z*<br>*z* ~0.167	½<br>0<br>0	*x*<br>1-*x*<br>*z*<br>*x* ~0.833<br>*z* ~0.667

↓ *t*3: $a_4 = 2a_3 + b_3$, $b_4 = b_3$, $c_4 = c_3$

Monoclinic
*C*2/*m* (12)
a_4 = 9.95 Å
b_4 = 5.74 Å
c_4 = 7.03 Å
β = 90°

Ba: 2*a*<br>2/*m*	Ba: 4*i*<br>4*m*	Co: 2*c*<br>2*m*	Co: 4*i*<br>*m*	O: 2*b*<br>2/*m*	O: 4*e*<br>-1	O: 4*i*<br>*m*	O: 8j<br>1
0<br>0<br>0	*x*<br>0<br>*z*<br>*x* ~0.833<br>*z* ~0.667	0<br>0<br>½	x<br>0<br>z<br>x ~ 0.333<br>z ~ 0.833	0<br>½<br>0	¼<br>¼<br>0	x<br>0<br>z<br>x ~ 0.833<br>z ~ 0.333	x<br>y<br>z<br>x ~ 0.417<br>y ~ 0.750<br>z ~ 0.667

↓ *k*2: $a_5 = a_4$, $b_5 = b_4$, $c_5 = c_4$, β = 90°

Monoclinic
*P*2$_1$/*m* (11)
a_4 = 9.95 Å
b_4 = 5.74 Å
c_4 = 7.03 Å
β = 90°

Ba: 2*e*<br>*m*	Ba: 2*e*<br>*m*	Ba: 2*e*<br>*m*	Co: 2*e*<br>*m*	Co: 2*e*<br>*m*	Co: 2*e*<br>*m*	O: 2*e*<br>*m*	O: 2*a*<br>-1	O: 2*b*<br>-1	O: 2*e*<br>*m*	O: 2*e*<br>*m*	O: 4f<br>1	O: 4f<br>1
x<br>¼<br>z<br>*x* ~0.250<br>*z* ~0.000	x<br>¼<br>z<br>*x* ~0.917<br>*z* ~0.667	x<br>¼<br>z<br>*x* ~0.583<br>*z* ~0.333	x<br>¼<br>z<br>x ~0.250<br>z ~0.500	x<br>¼<br>z<br>x ~ 0.917<br>z ~ 0.167	x<br>¼<br>z<br>x ~ 0.583<br>z ~ 0.833	0<br>¼<br>z<br>z ~ 0.833	0<br>0<br>0	½<br>0<br>0	x<br>¼<br>z<br>x ~ 0.417<br>z ~ 0.667	x<br>¼<br>z<br>x ~ 0.083<br>z ~ 0.333	x<br>y<br>z<br>x ~ 0.667<br>y ~ 0.000<br>z ~ 0.667	x<br>y<br>z<br>x ~ 0.833<br>y ~ 0.000<br>z ~ 0.333

Figure S5: Symmetry relationship showing the similarity between the cubic aristotype perovskite structure and the monoclinically distorted of perovskite type $BaCoO_{2.67}$.

Table S1: Ideal atomic coordinates for a pseudocubic perovskite within space group $P2_1/m$.

Atom	Wyckoff	*x*	*y*	*z*
Ba1	2*e*	0.250	¼	0.000
Ba2	2*e*	0.917	¼	0.667
Ba3	2*e*	0.583	¼	0.333
Co1	2*e*	0.250	¼	0.500
Co2	2*e*	0.917	¼	0.167
Co3	2*e*	0.583	¼	0.833
O1	2*e*	0.750	¼	0.000
O2	2*b*	½	0	0
O3	2*e*	0.417	¼	0.667
O4	2*e*	0.083	¼	0.333
O5	4*f*	0.667	0.000	0.667
O6	4*f*	0.833	0.000	0.333
O7	2*a*	0	0	0

Table S2: Refined atomic coordinates for $BaCoO_{2.67}$. O7- indicates the O-vacancy position. The red text indicates the coordinates symmetrically restricted. Rietveld refinement of NPD and XRD data were recorded at room temperature. Standard deviations given are numerical standard deviations from the refinements and do not represent interval of trust.

Atom	Wyckoff	*x*	*y*	*z*
Ba1	2*e*	0.2723(7)	¼	−0.0173(10)
Ba2	2*e*	0.9145(6)	¼	0.7191(8)
Ba3	2*e*	0.5683(7)	¼	0.3447(10)
Co1	2*e*	0.2681(16)	¼	0.485(2)
Co2	2*e*	0.8814(12)	¼	0.2200(18)
Co3	2*e*	0.581(2)	¼	0.837(3)
O1	2*e*	0.7541(15)	¼	0.035(3)
O2	2*b*	½	0	0
O3	2*e*	0.4194(17)	¼	0.666(3)
O4	2*e*	0.0362(12)	¼	0.131(2)
O5	4*f*	0.6804(12)	0.022(2)	0.6874(19)
O6	4*f*	0.8478(11)	−0.006(2)	0.3768(15)
O7-vacancy	2*a*	0	0	0

Table S3: Ab initio *optimized atomic coordinates for $BaCoO_{2.67}$ with U_{eff} = 4.5 eV.*

Atom	Wyckoff	*x*	*y*	*z*
Ba1	2*e*	0.272	¼	−0.009
Ba2	2*e*	0.912	¼	0.714
Ba3	2*e*	0.569	¼	0.338
Co1	2*e*	0.276	¼	0.501
Co2	2*e*	0.874	¼	0.228
Co3	2*e*	0.586	¼	0.838
O1	2*e*	0.755	¼	0.031
O2	2*b*	½	0	0
O3	2*e*	0.416	¼	0.661
O4	2*e*	0.032	¼	0.144
O5	4*f*	0.674	0.001	0.681
O6	4*f*	0.845	−0.009	0.372
O7-vacancy	2a	0	0	0

Table S4: (Experiment) Refined lattice parameters for $BaCoO_{2.67}$ after Rietveld refinements. For the refinements GOF (NPD+XRD) of 1.073 was observed along with the R_{Bragg} value of 7.927 % and 0.664 % for Bank1 and XRD respectively. (Ab initio) Ab initio optimized lattice parameters of G-AFM $P2_1/m$ $Ba_3Co_3O_8$ = $BaCoO_{2.67}$ with U_{eff} = 4.5 eV.

	a **(Å)**	***b*** **(Å)**	***c*** **(Å)**	***β*** **(°)**
**Experiment**	10.1717(1)	5.6038(1)	6.9248(1)	91.469(11)
Ab initio **(U_{eff} = 4.5 eV)**	10.260	5.639	6.999	91.884

Table S 5: Refined bond distances between cations and anions for $BaCoO_{2.67}$ at room temperature.

Cation	Anion	Distance (Å)	Cation	Anion	Distance (Å)
**Ba1**	O4 (x 1)	2.596(5)	**Co1**	O3 (x 1)	1.890(8)
	O3 (x 1)	2.721(6)		O6 (x 2)	2.033(6)
	O2 (x 2)	2.739(3)		O5 (x 2)	2.041(6)
	O5 (x 2)	2.805(5)			
	O1 (x 2)	2.8176(6)			
	O6 (x 2)	3.010(5)			
**Ba2**	O5 (x 2)	2.750(5)	**Co2**	O4 (x 1)	1.733(7)
	O1 (x 1)	2.758(5)		O6 (x 2)	1.801(5)
	O6 (x 2)	2.858(5)		O1 (x 1)	1.824(8)
	O6 (x 2)	2.875(4)			
	O4 (x 2)	3.0209(19)			
	O4 (x 1)	3.077(5)			
**Ba3**	O3 (x 1)	2.702(7)	**Co3**	O5 (x 2)	1.958(7)
	O3 (x 2)	2.8057(3)		O2 (x 2)	1.971(6)
	O2 (x 2)	2.856(4)		O3 (x 1)	2.038(10)
	O5 (x 2)	2.865(5)		O1 (x 1)	2.234(9)
	O1 (x 1)	2.909(6)			
	O5 (x 2)	2.951(5)			
	O6 (x 2)	3.153(5)			

Table S6: Bond valence sum (BVS) for the different crystallographic sites in the monoclinic $BaCoO_{2.67}$ with the global instability index of 0.3125.

Site	BVS	Site	BVS	Site	BVS
**Ba1**	2.5700	**Co1**	2.3573	**O1**	1.9560
**Ba2**	2.0711	**Co2**	3.3658	**O2**	2.0302
**Ba3**	2.3689	**Co3**	2.7675	**O3**	2.1882
				**O4**	1.8072
				**O5**	1.8637
				**O6**	1.8957

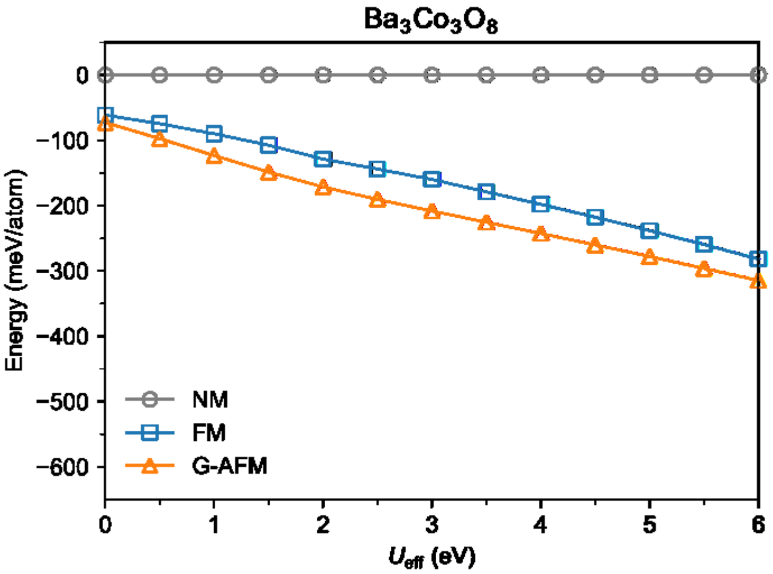

Figure S6: *Ab initio* computed energies of $Ba_3Co_3O_8$ = $BaCoO_{2.67}$ as a function of U_{eff}. For each U_{eff}, the energy of the NM state is set as the reference.

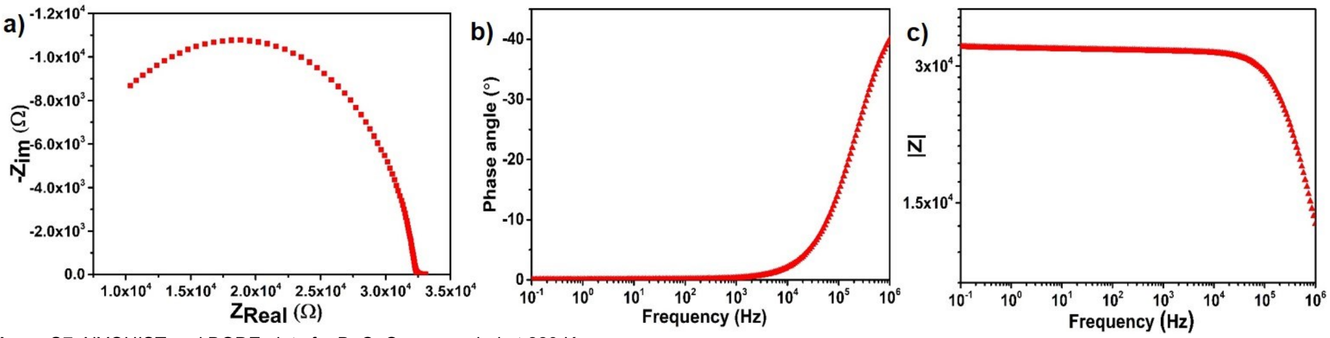

Figure S7: NYQUIST and BODE plots for $BaCoO_{2.67}$ recorded at 228 K.

4 Characterization of $Ba_{0.5}Sr_{0.5}Co_{0.8}Fe_{0.2}O_{3-x}$

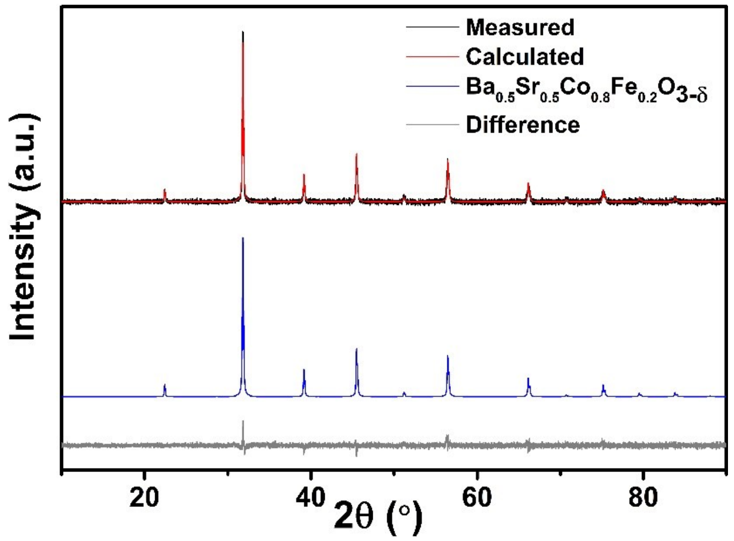

Figure S8: Rietveld fit of the BSCF powders synthesized from the nebulized spray pyrolysis technique.

As mentioned previously, BSCF powders were synthesized via the NSP method. The as prepared powders where then heat treated at 1073 K for 6 hours in argon atmosphere. Figure S8 shows the Rietveld fit of the room temperature X-ray diffraction pattern recorded for the phase pure BSCF powders. In agreement with the previous reports [13], BSCF was found to crystallize in a cubic structure within the space group *Pm*−3*m* and the lattice parameter of 4.0030(1) Å was determined from the refinements. The powders were then subjected to iodometric titrations to determine the average oxidation state for the *B* site cation (Co/Fe) which was calculated to be +2.75(1). Recently, we have shown for the $BaFe_{1-x}Co_xO_{3-y-\delta}(OH)_y$ [1] series synthesized via NSP method that Fe is mainly present in the +3 oxidation state irrespective of the Fe to Co ratio. In this series Co is mainly present in the mixed valent +2/+3 oxidation state for Co-rich samples [1, 14]. This applies also for Fe in BSCF [15]. Herein the Fe is predominantly in the +3 oxidation state whereas Co is present in the mixed oxidation state of +2/+3. To verify this, we have prepared a series of compounds with the composition $Ba_{0.5}Sr_{0.5}Co_{1-x}Fe_xO_{3-\delta}$ and studied them for their oxygen vacancy concentration via iodometric titrations. We observed that for Fe rich composition (x = 1), the average oxidation state of B site was +3, which is similar to what we found for the $BaFe_{1-x}Co_xO_{3-y-\delta}(OH)_y$ [1] series. Therefore, in case of BSCF, our assumption of +3 oxidation state for Fe is valid and changes in the oxidation states would be as a result of lowering of oxidation number for Co. Further, scanning electron micrographs of BSCF, intermediate $BaCoO_{2.46}$ and $BaCoO_{2.67}$ were recorded and are shown in Figure S9. It is clear that particle size of the powders synthesized via the NSP route are smaller than the particles synthesized via the solid state route.

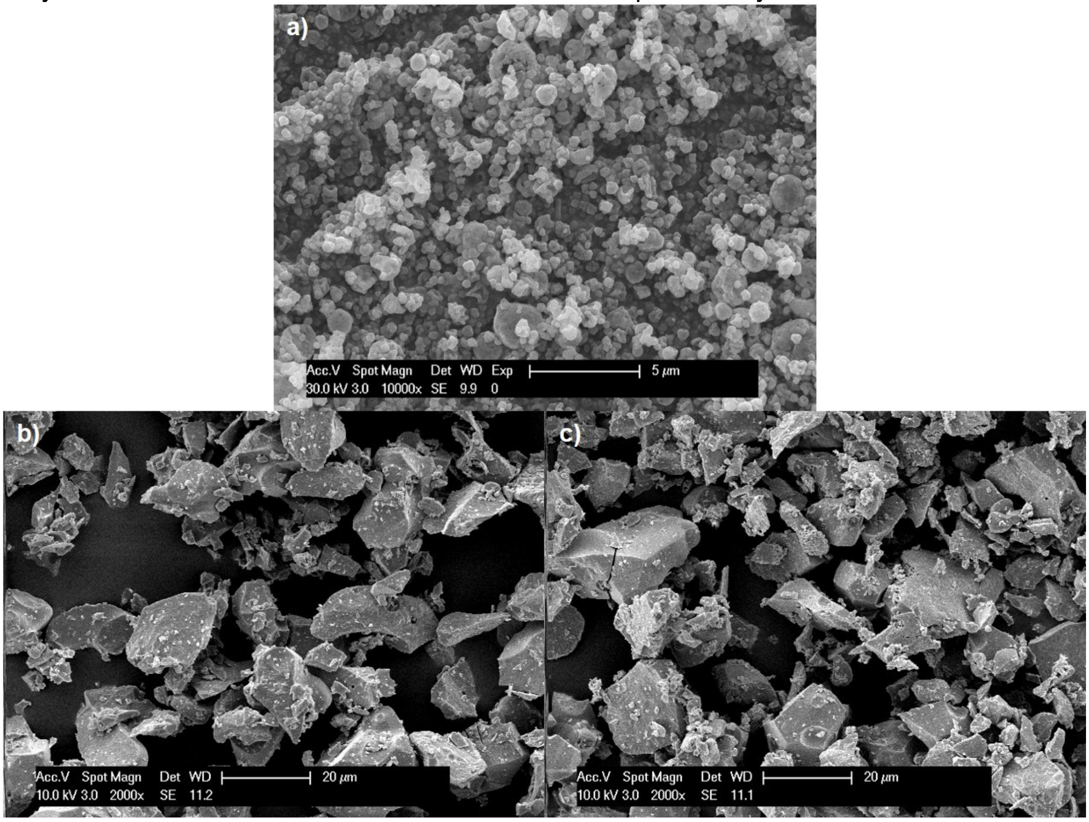

Figure S9: SEM micrographs recorded for 1)BSCF synthesized via NSP method b)intermediate phase $BaCoO_{2.46}$ and c) $BaCoO_{2.67}$.

The bifunctional catalytic activity towards ORR and OER were measured and presented in the main article. However, in order to determine the intrinsic catalytic activity towards the ORR and OER, we also carried out the measurements without the carbon additive and the results are presented in Figure S10. Interestingly BSCF shows better intrinsic catalytic activity compared to that of the title compound. This enhanced activity could be as a result of increased number of density of states (DOS) near the fermi lever for BSCF as compared to $BaCoO_{2.67}$ (Figure S11) which is beneficial towards the intrinsic catalytic activity. Nonetheless, the catalytic activity in the absence of carbon is still low and signifies the role of carbon additive towards improvement of electronic conductivity. Once carbon is added, both compounds show a similar order of magnitude regarding their performance, with $BaCoO_{2.67}$ showing a significant improvement over BSCF in ORR and OER.

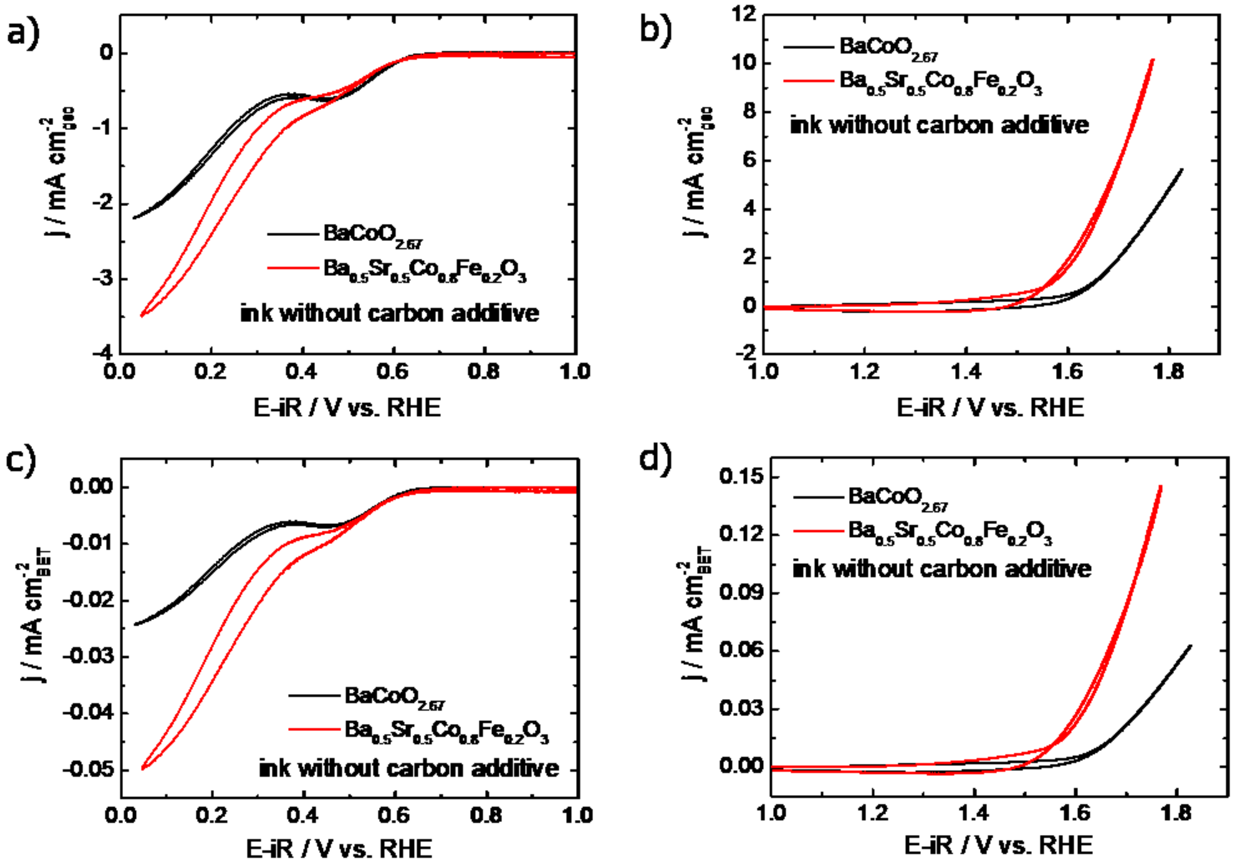

Figure S10: Comparison of as-measured geometric current densities towards a) ORR and b) OER and after normalization to the BET specific surface area of the samples c) for ORR and d) OER activity. In all graphs $BaCoO_{2.67}$ and BSCF measured in 0.1M KOH without the addition of conducting carbon are displayed.

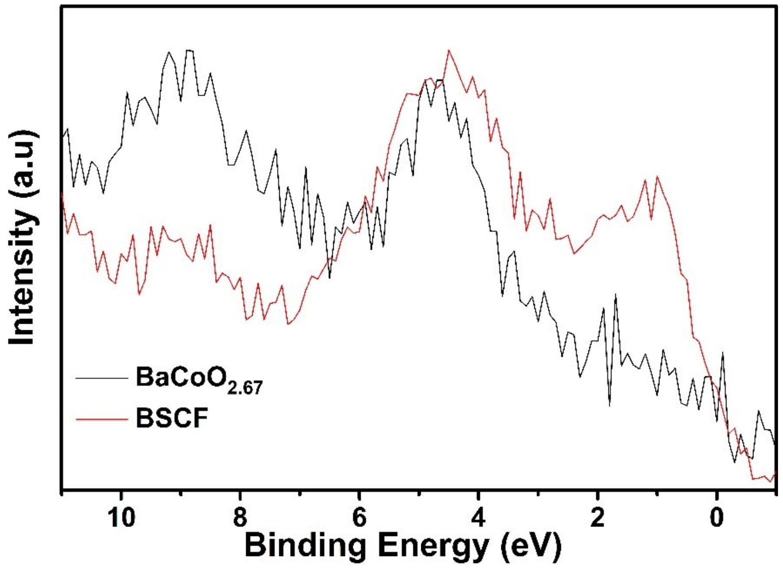

Figure S11: Valence band comparison of $BaCoO_{2.67}$ and BSCF.

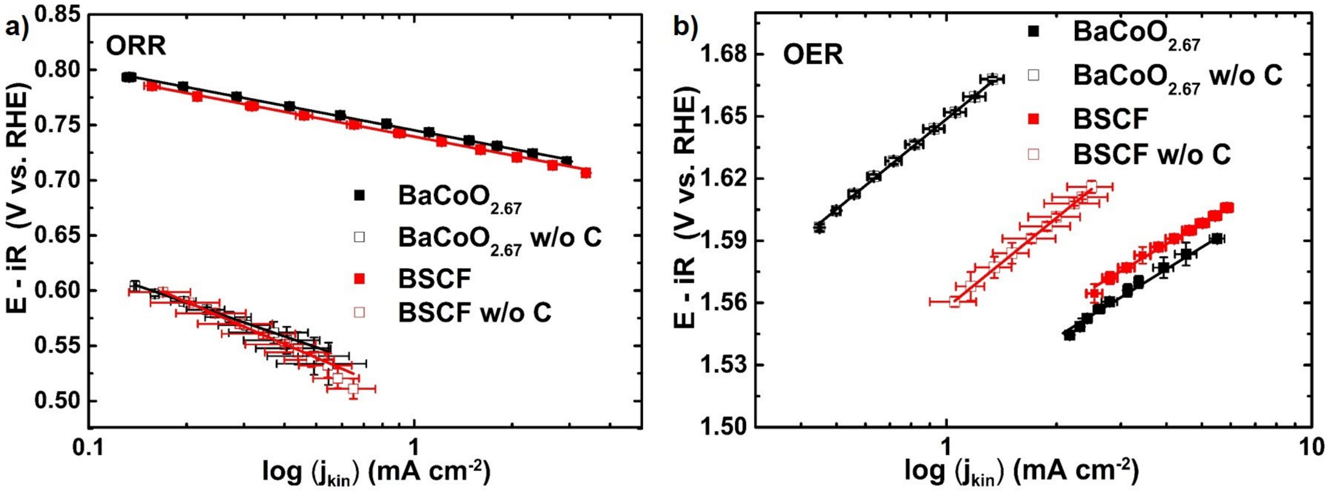

Figure S12: Comparison of a) ORR Tafel slopes and b) OER Tafel slopes for $BaCoO_{2.67}$ and BSCF measured with and without carbon.

Table S7: Values of Tafel slopes for $BaCoO_{2.67}$ and BSCF.

Sample	OER		ORR	
	With Carbon	Without Carbon	With Carbon	Without Carbon
BSCF	105	144.4	56	126
$BaCoO_{2.67}$	97.8	144.7	55	101

References

[1] A. I. Waidha, L. Ni, J. Ali, M. Lepple, M. Donzelli, S. Dasgupta, S. Wollstadt, L. Alff, U. I. Kramm, O. Clemens, *J. Mater. Chem. A* **2020**, *8*, 616-625.
[2] *Topas V4.2, General profile and structure analysis software for powder diffraction data, User's Manual*, Bruker AXS, Karlsruhe, Germany, **2008**.
[3] R. W. Cheary, A. A. Coelho, J. P. Cline, *J Res Natl Inst Stand Technol* **2004**, *109*, 1-25.
[4] D. Johnson, *Inc., Southern Pines, NC* **2002**, *200*.
[5] E. O. Wollan, W. C. Koehler, *Physical Review* **1955**, *100*, 545-563.
[6] P. E. Blochl, *Phys Rev B Condens Matter* **1994**, *50*, 17953-17979.
[7] J. P. Perdew, K. Burke, M. Ernzerhof, *Phys Rev Lett* **1996**, *77*, 3865-3868.
[8] G. Kresse, J. Furthmuller, *Computational Materials Science* **1996**, *6*, 15-50; bG. Kresse, J. Furthmuller, *Phys Rev B Condens Matter* **1996**, *54*, 11169-11186.
[9] G. Kresse, D. Joubert, *Phys Rev B* **1999**, *59*, 1758-1775.
[10] M. Methfessel, A. T. Paxton, *Phys Rev B Condens Matter* **1989**, *40*, 3616-3621.
[11] S. L. Dudarev, G. A. Botton, S. Y. Savrasov, C. J. Humphreys, A. P. Sutton, *Phys Rev B* **1998**, *57*, 1505-1509.
[12] V. Pardo, P. Blaha, M. Iglesias, K. Schwarz, D. Baldomir, J. E. Arias, *Phys Rev B* **2004**, *70*, 144422.
[13] M. G. Sahini, J. R. Tolchard, K. Wiik, T. Grande, *Dalton Trans.* **2015**, *44*, 10875-10881.
[14] A. I. Waidha, M. Lepple, K. Wissel, A. Benes, S. Wollstadt, P. R. Slater, A. D. Fortes, O. Clemens, *Dalton Trans.* **2018**, *47*, 11136-11145.
[15] J. Suntivich, K. J. May, H. A. Gasteiger, J. B. Goodenough, Y. Shao-Horn, *Science* **2011**, *334*, 1383-1385.
